# Point-of-care diagnostics for sexually transmitted infections among migrants in Greece

**DOI:** 10.5588/pha.23.0022

**Published:** 2024-03-01

**Authors:** C. Tsorou, A. Williams, W. van den Boogaard, N. Staderini, E. Repetto, A. Terzidis, E. Pikoulis

**Affiliations:** ^1^ Médecins Sans Frontières (MSF), Operational Centre Geneva, Mission Greece, Athens,; ^2^ Disaster Medicine, School of Medicine, National and Kapodistrian University of Athens, Athens, Greece,; ^3^ MSF, Luxembourg Operational Research (LuxOR) Unit, Operational Centre Brussels, Brussels,; ^4^ MSF, Middle East Medical Unit (MEMU), Operational Centre Brussels, Belgium,; ^5^ MSF, Operational Centre Geneva Medical Department, Geneva, Switzerland;; ^6^ Infectious Diseases Unit, Saint Pierre University Hospital, Brussels, Belgium

**Keywords:** GeneXpert CT/NG test, *Chlamydia Trachomatis*, *Neisseria gonorrhoeae*, antimicrobial resistance, SORT IT, Structured Operational Research and Training Initiative

## Abstract

**SETTING:**

Sexually transmitted infections (STIs) can impact individuals of any demographic. The most common pathogens causing STIs are *Chlamydia trachomatis*, *Neisseria gonorrhea* and *Trichomonas vaginalis*; these can be treated with specific antibiotics.

**OBJECTIVE:**

To compare the GeneXpert CT/NG test-and-treat algorithm to the syndromic approach algorithm and their impact on antibiotic prescription for gonorrhoea and chlamydia STIs.

**DESIGN:**

A retrospective observational study on women aged ≥18 years who accessed the Médecins Sans Frontières Day Care Centre in Athens with complaints related to urogenital infections between January 2021 and March 2022. Women with abnormal vaginal discharge, excluding clinically diagnosed candidiasis, were eligible for Xpert CT/NG testing.

**RESULTS:**

Of the 450 women who accessed care, 84 were eligible for Xpert CT/NG testing, and only one was positive for chlamydia, therefore resulting in saving 81 doses of ceftriaxone and azithromycin, and 19 doses of metronidazole. The cost of Xpert CT/NG testing, including treatment was €4,606.37, while full antibiotic treatment would have costed €536.76.

**CONCLUSION:**

The overall cost of the Xpert CT/NG test-and-treat algorithm was higher than the syndromic approach. However, quality of care should be weighed against the potential benefits of testing and syndromic treatment to determine the best option for each patient; we therefore advocate for decreasing the costs.

## Abstract (fr)

**CONTEXTE:**

Les infections sexuellement transmissibles (STI, pour l’anglais « sexually transmitted infections ») touchent tous les individus. Les agents pathogènes les plus courants à l'origine des STI sont *Chlamydia trachomatis*, *Neisseria gonorrhea* et *Trichomonas vaginalis*, et ils peuvent être traités avec des antibiotiques spécifiques.

**OBJECTIF:**

Comparer l'algorithme test-and-treat du GeneXpert CT/NG à l'algorithme de l'approche syndromique et leur impact sur la prescription d'antibiotiques pour les STI à gonorrhée et à chlamydia.

**MÉTHODE:**

Une étude observationnelle rétrospective sur les femmes âgées de ≥18 ans qui ont accédé au centre de soins de jour de Médecins Sans Frontières à Athènes avec des plaintes relatives aux infections urogénitales entre janvier 2021 et mars 2022. Les femmes présentant des pertes vaginales anormales, à l'exclusion des candidoses cliniquement diagnostiquées, étaient éligibles pour le test GeneXpert CT/NG.

**RÉSULTATS:**

Sur les 450 femmes qui ont eu accès aux soins, 84 étaient éligibles au test GeneXpert CT/NG et une seule était positive à la chlamydia, ce qui a permis d'économiser 81 doses de ceftriaxone et d'azithromycine, et 19 doses de métronidazole. Le coût du test GeneXpert CT/NG, traitement compris, s'est élevé à €4 606,37, tandis qu'un traitement antibiotique complet aurait coûté €536,76.

**CONCLUSION:**

Le coût global de l'algorithme GeneXpert CT/NG test-and-treat était plus élevé que celui de l'approche syndromique. Cependant, la qualité des soins doit être mise en balance avec les avantages potentiels des tests et du traitement syndromique afin de déterminer la meilleure option pour chaque patient, et nous plaidons par conséquent en faveur d'une diminution des coûts.

Greece is one of the main entry points for people currently coming from Syria, Afghanistan, Iraq and Pakistan moving towards the European Union (EU), of whom at least 16,600 have been stranded in Greece since the implementation of the EU-Turkey deal.^1^ The conditions of migration, the procedures of integration into a new society, the social status of females and health regulations are all factors that affect a woman’s wellbeing, in particular issues regarding sexual and reproductive health (SRH).^2–4^

Sexually transmitted infections (STIs) can affect individuals of any demographic. Refugees, migrants and residents from low-resource settings are at higher risk of STIs.^2,5^ The most common curable STIs are gonorrhoea (*Neisseria gonorrhoeae)*, chlamydia (*Chlamydia trachomatis)* and trichomoniasis (*Trichomonas vaginalis)*, all of which cause cervicitis in women and urethritis in both men and women.^6^ The WHO estimated that in 2020, 374 million new infections were recorded due to these causes. Other infections causing similar symptomatology include candidiasis and bacterial vaginosis. Of great concern is the increasingly antibiotic resistance of *N. gonorrhoeae*. Following the spread of gonococcal fluoroquinolone resistance, cephalosporins have been the foundation of recommended treatment guidelines for gonorrhoea.^7,8^

The syndromic approach for treatment of STIs is widely used, and is included in Médecins Sans Frontières (MSF) clinical guidelines as standard of care since adequate laboratory infrastructure is lacking in most low-resource settings. The limitations of this approach have been well-documented and are, among others, the inability to diagnose asymptomatic infections, the poor predictive values for the diagnostic capacity of identifying and confirming infectionsand the overprescription of antibiotics.^6,9,10^ Appropriate antibiotic prescription is the essence of antibiotic stewardship and is valid for any infectious syndrome, including STIs.^6,11^ In order to be able to maximise targeted antibiotic prescription, access to specific diagnostics is required. This implies the need for the development and implementation of new approaches.^12,13^

The WHO recommends the development and adoption of rapid testing technologies for STIs,^14^ such as the GeneXpert CT/NG System (Cepheid, Sunnyvale, CA, USA) for the detection of *C. trachomatis* (CT) and *N. gonorrhoea*e (NG) in one cartridge with high sensitivity and specificity.^15^ MSF introduced the Xpert CT/NG testing at their Athens Day Care Centre (DCC), an outpatient department for refugees, in its SRH services in January 2021. With Xpert CT/NG testing, healthcare providers are able to deliver results within 90 min, which allows for targeted antibiotic treatment to be prescribed on the same day. A downside is that the cartridges for running GeneXpert CT/NG are very expensive; in many resources-limited settings, this is still therefore not an option that can be easily implemented as standard of care.

This study focused on symptomatic female patients seeking SRH services at the MSF DCC in Athens between January 2021 and March 2022. The primary objective was to determine whether the implementation of Xpert CT/NG testing resulted in improved, rational antibiotic prescriptions for these STIs. Second, costs of saved antibiotics were compared with the costs of Xpert CT/NG testing.

## METHODS

### Study settings

The MSF DCC, located in the centre of Athens, serves those who do not have access to the public healthcare system. Services provided at the DCC include SRH consultations, including care for survivors of sexual and gender-based violence (SGBV), mental health, and non-communicable diseases (NCDs), and integrated travel medicine services (TMS). Complementary multidisciplinary support is provided alongside the primary care services, including socio-legal services and health promotion. In 2020, MSF provided more than 7,000 SRH consultations.^9^

### Design, study population and period

This was a retrospective observational study using routinely collected data from women aged ≥18 years accessing SRH services at the MSF DCC who presented with complaints related to urogenital infections as part of our study's inclusion criteria between January 2021 to March 2022.

### Routine clinical care procedure

The MSF DCC uses a guided decision-making algorithm for the management of STIs (Figure).^16,17^ During the consultation, the midwife collects complaints and sexual history from the patient, manages the clinical examination and evaluates risk factors accordingly. In case of pain or lesions of the urogenital-anal area, the midwife inspects the urethral meatus, the anal margin and the external genital organs for the presence of discharge, ulceration, mass, bubolephadenopathy, etc. In case of STI suspicion in women, speculum examination, vaginal examination and bimanual palpation are conducted for establishing a syndromic diagnosis as precisely as possible.

**FIGURE.**
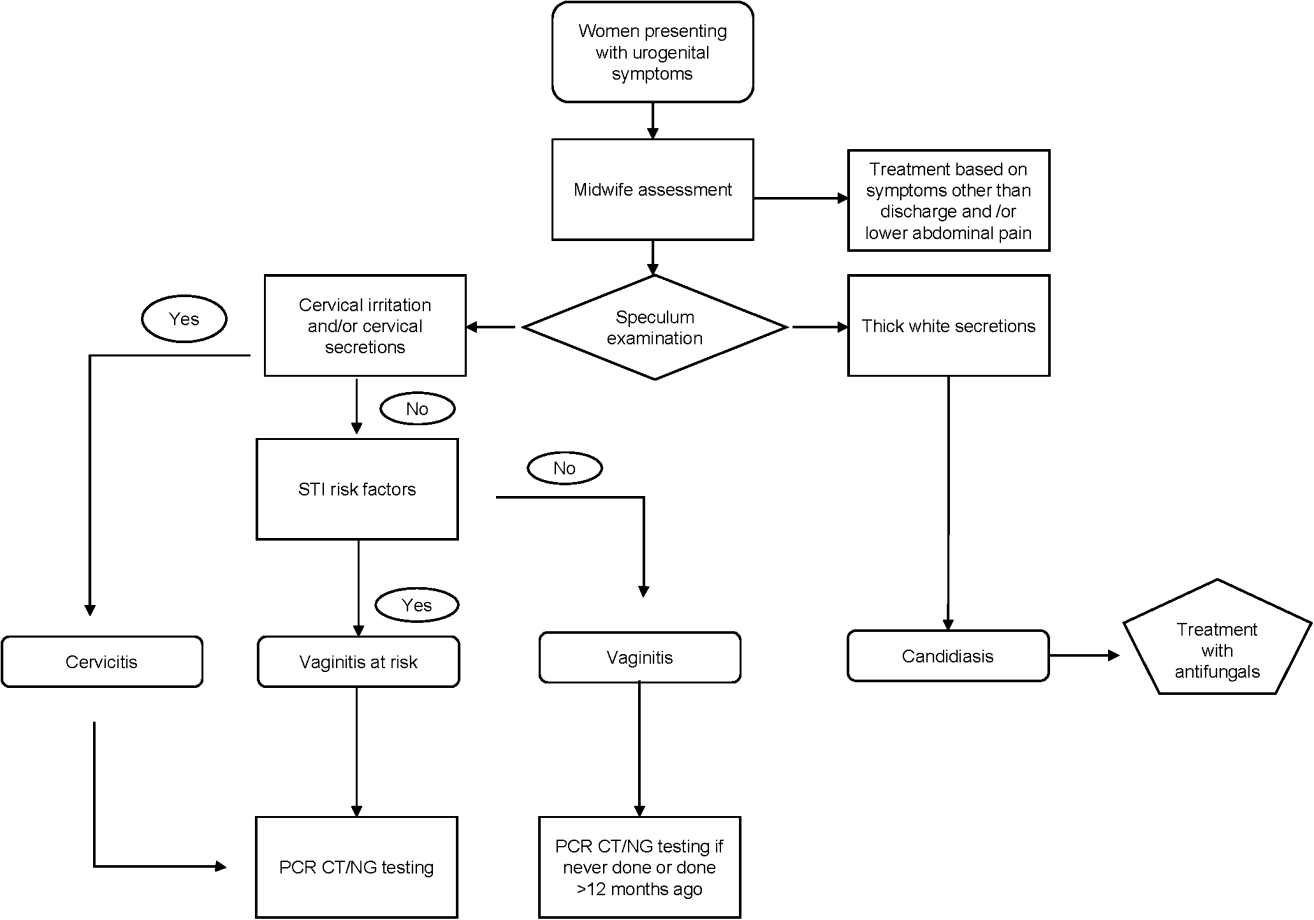
Decision-making algorithm as per MSF guidelines. Risk factors include new partner or >1 partners in the last 3 months, oral/anal/genital complaint and/or dysouria in a man aged <30 years or a woman aged <25 years, and/or treated STI/genital/urethral/anal discharge present in the partner. STI = sexually transmitted infection; PCR = polymerase chain reaction; CT *= C. trachomatis*; NG = *N. gonorrhoeae*; MSF = Médecins Sans Frontières.

When a patient has cervical irritation and/or secretions from the cervix that is not consistent with candidiasis, a swab sample of the discharge is taken, and an Xpert CT/NG test is performed. Results are conveyed to the women on the same day, and treatment is provided to the patient free of charge. All the patients are treated according to their symptoms following internal MSF guidelines as described below (Table 1).^16,17^

**TABLE 1.** Treatment for cervicitis and/or vaginitis, syndromic vs GeneXpert CT/NG test-and-treat approach/algorithm according to MSF guidelines.^17^

Syndromic approach treatment	Treatment according to GeneXpert result
Azithromycin 1 g SD *and* Ceftriaxone 250 mg IM SD *and* Metronidazole 2 g SD	If NG-positive: ceftriaxone 250 mg IM SD + azithromycin 1 g SD If CT-positive: azithromycin 1 g SD If negative for both CT and NG: metronidazole 2 g SD
Partner treatment: same as given to patient	

NG = *N. gonorrhoeae* CT *= C. trachomatis*; MSF = Médecins Sans Frontières; SD = single-dose; IM = intramuscular.

### Testing procedures

The midwife prepares the GeneXpert CT/NG cartridge and runs the sample, in accordance with the manufacturers’ guidelines and training received from Cepheid representatives,^10^ reads the results, and enters these in the patient records and into an electronic clinical database. External quality controls to monitor the extraction, amplification and detection of the Cepheid GeneXpert CT/NG assay equipment are run by a Cepheid representative every 6 months.

### Data analysis

Data were retrieved from the clinical database. The proportion of overtreatment using the syndromic approach was calculated by calculating the difference between the number of antibiotic treatments given after GeneXpert CT/NG test results compared to the number of antibiotic treatments which would have been given if no GeneXpert CT/NG test was conducted. To calculate the costs involved in the syndromic approach compared to the GeneXpert STI algorithm, the number and cost of antibiotic treatments which would have been given according to syndrome guidelines was compared to the number and cost of antibiotic treatments given after Xpert CT/NG test results. This difference was then compared to the costs involved in GeneXpert testing.

### Ethics

This study was approved by the National Kapodistrian University of Athens, Athens, Greece, following a review by the Scientific Board of the University Hospital Attiko, Athens, Greece (ID: 312); and the MSF Ethical Review Board (ERB), Paris, France (ID: 2161). The need for individual informed consent was waved by the MSF ERB.

## RESULTS

During the study period, 450 adult women visited the SRH services of the MSF DCC, presenting with complaints related to urogenital infections; 98 (21.8%) of these were between 18 and 25 years old. The most common region of origin was Asia (*n* = 240, 53%). The main initial complaint was discharge (*n* = 178, 40%) and the most common syndromic diagnosis from the healthcare provider was vaginal candidiasis (*n* = 236, 52%) (Table 2). Of the 84 “at risk” patients with cervicitis and vaginitis who were tested using Xpert CT/NG, 24 (29%) were 18–25 years old, and none were aged >50 years (Table 3). One (1.2%) of these 84 women had a positive chlamydia Xpert result.

**TABLE 2.** Demographics and clinical characteristics of female patients that were consulted in the SRH services and presented complaints relevant to urogenital infections in MSF Athens DCC between January 2021 to March 2022.

Characteristic	*n* (%)
Age range	
18–25	98 (21.8)
26–49	264 (57.5)
≥50	7 (1.6)
Missing data	81 (18)
Geographic origin	
Africa	177 (39.3)
Asia	240 (53.3)
Other	33 (7.3)
Initial patient complaint	
Discharge	178 (39.5)
Dysuria	15 (3.3)
Genital itchiness	130 (28.8)
Skin lesions	2 (0.4)
Lower abdominal pain	115 (25.5)
Other	10 (2.2)
Main syndromic diagnosis	
Cervicitis	37 (8.2)
Vaginitis	106 (23.5)
Trichomoniasis	3 (0.6)
Genital herpes	1 (0.2)
Upper genitalia infection	2 (0.4)
Urinary tract infection	13 (2.8)
Urogenital ulceration	4 (0.8)
Vaginal candidiasis	236 (52.4)
Normal	33 (7.3)
Other	4 (0.8)
Missing data	11 (2.4)

SRH = sexual and reproductive health; DCC = day care centre.

**TABLE 3.** Demographic and clinical characteristics of female patients who presented with discharge or lower abdominal pain and a GeneXpert CT/NG test result in MSF Athens DCC, January 2021–March 2022.

Characteristics	*n* (%)
Age range	
18–25	24 (28.5)
26–49	60 (71.4)
≥50	0 (0)
Geographic origin	
Asia	62 (73.8)
Africa	21 (25)
Other	1 (1.1)
GeneXpert test	
CT–/NG–	82 (97.6)
CT+/NG+	0 (0)
CT–/NG+	0 (0)
CT+/NG–	1 (1.2)
Non-applicable	1 (1.1)

MSF = Médecins Sans Frontières; DCC = day care centre; NG = *N. gonorrhoeae*; CT *= C. trachomatis*; – = negative; + = positive.

The impact of Xpert CT/NG testing on the number of antibiotics prescribed was assessed; results showed that fewer antibiotic treatments were given compared to the syndromic approach (Table 4). With the syndromic approach, all 84 women would have received antibiotic treatment regimens (ceftriaxone 250 mg intramuscular, azithromycin 1 g sub-dermal [SD] and metronidazole 2 g SD). However, with the implementation of GeneXpert, only 81 ceftriaxone treatments were given, resulting in savings of €489.07.

**TABLE 4.** Assessment of the impact of the implementation of GeneXpert CT/NG testing on the prescription of antibiotics in female patients presenting with cervitis or vaginitis in MSF Athens DCC, January 2021–March 2022 (*n* = 84).

	If no GeneXpert test		GeneXpert test performed			
	Antibiotics	Cost	Antibiotics	Cost	Antibiotics	Cost
Antibiotic treatments	*n*	(€)	given	(€)	saved	(€)
Ceftriaxone 250 mg IM SD	84	369.6	3	13.2	81	356.4
Azithromycin 1 g PO SD	84	128.52	3	4.6	81	123.3
Metronidazole 2 g PO SD	84	38.64	65	29.8	19	8.4
Total cost, €		536.76		47.6		489.07

CT *= C. trachomatis*; NG = *N. gonorrhoeae*; MSF = Médecins Sans Frontières; DCC = day care centre; IM = intramuscular; SD = single-dose; PO = taken by mouth.

The total cost of antibiotics for the 450 women who received treatment using the syndromic approach was €536.76. If all 84 women had been treated using this approach, the cost would have been €47.69. However, the implementation of the Xpert testing saved 81 doses of ceftriaxone, which resulted in savings of €489.07. On the other hand, the cost of testing for the 84 women was €4,558.68, which includes the price of consumables used (€54.27/test). When the cost of testing was added to the targeted antibiotic costs, the total cost per patient amounted to €54.83 (Table 4). It is worth noting that despite the negative test results, three women still received antibiotics. Therefore, only 81 doses of treatments were actually saved.

## DISCUSSION

Among the 450 refugee women presenting with complaints related to urogenital infections in the MSF’s Athens DDC, 84 met the criteria for GeneXpert Ct/NG testing, and only one patient tested positive for chlamydia and was given oral azithromycin 1 g SD antibiotic treatment. If all had been treated according to the syndromic approach, 83 women would have received unnecessary antibiotics. While the cost of antibiotics saved was certainly small when compared to the cost of testing these women using GeneXpert, this does raise some salient points for discussion in this era of increasing antimicrobial resistance (AMR).

The main symptoms presented were discharge and genital itchiness, and the most common syndromic diagnosis from the healthcare provider was vaginal candidiasis. This is reasonable, given that vaginal yeast infection is common in women. There are several risk factors that may increase the chances of developing a yeast infection, including the use of antibiotics besides birth control, diabetes, pregnancy, and a weakened immune system; however, those factors have not been analysed in this study.^11^

It was expected that we would see a higher number of positive CT/NG tests, as the population being served at the MSF’s Athens DCC face several challenges, including access to healthcare, high-risk sexual activities and sexual violence. Furthermore, these women come from places where STI transmission and infection risks are higher.^12,13,18–20^ Based on the results of this study, patients at the MSF DCC, and their partners, would have received unnecessary antibiotics had the syndromic approach been used.

While on the one hand antibiotic, over-prescription is common with the syndromic approach, there is also an underdiagnosis of asymptomatic cases who need antibiotic treatment in order to curb transmission and prevent long-term STI-related complications.^21^ While in communities with known high STI prevalence, it is appropriate to use the syndromic management approach, the test-and-treat approach is advised where prevalence is low or unknown in order to optimise antibiotic prescription.^22,23^ Clearly the risks and benefits of the syndromic approach should be weighed and calculated, especially for migrant populations living in developed countries.^24^

Point-of-care testing (POCT) should not only be adopted by MSF and other non-governmental organisations providing care for migrants, it should also be adopted by the Greek health system for all patients to reduce the time to treatment and the overuse of antibiotics. The newest guidelines from the Greek National Organization of Public Health recommend the use of nucleic acid amplification tests, but microbial culture is still used in general practice.^25^ Moreover, it is worth mentioning that in Greece, antibiotics could be bought over the counter without a medical prescription until 1 September 2020. This was common practice, as a lot of clinics waiting times were long, and the costs of consultations and tests were high.

As was expected, the cost of antibiotic treatments given after GeneXpert CT/NG testing was lower; however, the cost of using GeneXpert as standard practice was much higher. Therefore, when comparing costs in absolute terms, Xpert CT/NG may appear inefficient; however, this does not take into consideration the different direct and indirect costs. Investing in better access to diagnosis may involve high upfront costs such as purchasing medical equipment, training medical personnel and providing financial support for patients, but this does not take into account the costs associated with the increase in AMR related to overtreatment, especially that related to the control of *Neisseria gonorrohoea*,^26–29^when using the syndromic approach for STIs. Although the Xpert test does not provide information on the resistance pattern of the bacteria, it is important to note that the test specifically targets the bacteria and reduces the emergence of AMR, which is a crucial factor in preventing the spread of antibiotic resistance. This merits further research, including health economic evaluations.

It is important that the cost of GeneXpert CT/NG cartridges be reduced in order to enhance universal access to this diagnostic tool, especially in low-resourced settings. There is clearly a role for wide advocacy, such as the MSF Access Campaign,^30^ directed at the company(s) producing these tests to reduce the price as has been done for other tests and HIV treatments.^31^ As GeneXpert is widely used in low-resource settings for TB and HIV testing, an adaptation of the syndromic algorithm for STIs as described in this paper is feasible.

### Strengths and weaknesses

This study had some limitations. Not all women were included, but only those aged >18 years. During the study period, only one patient aged <18 years came for consultation. Men were not included as they were usually treated as the patient’s partner. The results of this study may not be representative of the migrant population at large. It is important to acknowledge that the results of this study are unexpected and contradictory to those of other studies. This may be due to several factors, such as the study's limited sample size or the unique characteristics of the population studied, particularly among migrants. Caution should be taken in generalising the results, or assuming that these are representative of the real-life situation or incidence of STI in this population.

As this study used routinely collected programme data, we cannot guarantee 100% data validation. Study strengths include the fact that this is the first study conducted in Greece, an EU country, that targets migrant populations.

## CONCLUSION

In limited access to healthcare settings, the use of the syndromic approach for STIs is the most common standard of care; however, in order to reduce overprescription of antibiotics, the inclusion of rapid testing is strongly advocated to improve quality of care and ensure targeted antibiotic treatment. Overall, this study shows the importance of implementing the adapted syndromic algorithm (including Xpert CT/NG testing) in high-risk populations to reduce the burden of disease and improve health outcomes in those populations. The cost-effectiveness of this approach should be emphasised, with potential long-term savings in healthcare costs, reduced risks of AMR and improved quality of life. Finally, there is a need for collaboration between healthcare providers, policymakers and the community to ensure the successful implementation of this adapted approach.
